# Comparing ChatGPT 4.0’s Performance in Interpreting Thyroid Nodule Ultrasound Reports Using ACR-TI-RADS 2017: Analysis Across Different Levels of Ultrasound User Experience

**DOI:** 10.3390/diagnostics15050635

**Published:** 2025-03-06

**Authors:** Katharina Margherita Wakonig, Simon Barisch, Leonard Kozarzewski, Steffen Dommerich, Markus Herbert Lerchbaumer

**Affiliations:** 1 Department of Otorhinolaryngology, Charité—Universitätsmedizin Berlin, Corporate Member of Freie Universität Berlin and Humboldt Universität zu Berlin, Campus Virchow Klinikum and Campus Charité Mitte, Charitéplatz 1, 10117 Berlin, Germany; 2 Department of Endocrinology, Diabetes and Metabolism, Charité—Universitätsmedizin Berlin, Corporate Member of Freie Universität Berlin and Humboldt Universität zu Berlin, Charitéplatz 1, 10117 Berlin, Germany; 3 Department of Radiology, Charité—Universitätsmedizin Berlin, Corporate Member of Freie Universität Berlin and Humboldt Universität zu Berlin, Charitéplatz 1, 10117 Berlin, Germany

**Keywords:** thyroid nodules, large language models, diagnostic accuracy, artificial intelligence, artificial hallucinations

## Abstract

**Background/Objectives:** This study evaluates ChatGPT 4.0’s ability to interpret thyroid ultrasound (US) reports using ACR-TI-RADS 2017 criteria, comparing its performance with different levels of US users. **Methods:** A team of medical experts, an inexperienced US user, and ChatGPT 4.0 analyzed 100 fictitious thyroid US reports. ChatGPT’s performance was assessed for accuracy, consistency, and diagnostic recommendations, including fine-needle aspirations (FNA) and follow-ups. **Results:** ChatGPT demonstrated substantial agreement with experts in assessing echogenic foci, but inconsistencies in other criteria, such as composition and margins, were evident in both its analyses. Interrater reliability between ChatGPT and experts ranged from moderate to almost perfect, reflecting AI’s potential but also its limitations in achieving expert-level interpretations. The inexperienced US user outperformed ChatGPT with a nearly perfect agreement with the experts, highlighting the critical role of traditional medical training in standardized risk stratification tools such as TI-RADS. **Conclusions:** ChatGPT showed high specificity in recommending FNAs but lower sensitivity and specificity for follow-ups compared to the medical student. These findings emphasize ChatGPT’s potential as a supportive diagnostic tool rather than a replacement for human expertise. Enhancing AI algorithms and training could improve ChatGPT’s clinical utility, enabling better support for clinicians in managing thyroid nodules and improving patient care. This study highlights both the promise and current limitations of AI in medical diagnostics, advocating for its refinement and integration into clinical workflows. However, it emphasizes that traditional clinical training must not be compromised, as it is essential for identifying and correcting AI-driven errors.

## 1. Introduction

Ultrasound (US) has long been regarded as an essential tool for the routine evaluation of thyroid pathologies due to its straightforward accessibility of the neck and the opportunity to immediately perform a US-guided fine needle aspiration (FNA) if histological workup is indicated [1,2]. The US is widely utilized by healthcare professionals, including otolaryngologists, radiologists, and endocrinologists, to identify and assess potential thyroid issues [3,4]. The rapid evolution of medical imaging technologies has markedly enhanced diagnostic accuracy and management strategies for various diseases, with thyroid nodules being a prime example [5]. The advent of high-resolution US has revolutionized the detection and evaluation of thyroid nodules, enabling clinicians to assess the risk of malignancy with greater precision [6]. To ensure accurate evaluation of thyroid nodules, the American College of Radiology (ACR) updated the Thyroid Imaging Reporting and Data System (TI-RADS) in 2017 [7]. The ACR-TI-RADS guidelines focus on five key aspects of a nodule: composition, echogenicity, shape, margins, and echogenic foci, providing a structured approach for risk stratification to determine whether a biopsy or follow-up examination is necessary [8]. Familiarity with these criteria is essential for all practitioners conducting thyroid US examinations, advocating for early integration of these guidelines into medical training to enhance diagnostic proficiency [9,10,11]. A detailed description of the nodule’s five critical characteristics in a structured US report allows for subsequent evaluation using the official ACR-TI-RADS criteria [9].

For inexperienced US users, the correct identification and description of a thyroid nodule can be overwhelming, particularly when it comes to real-time interpretation of the findings. While this poses no issue for more experienced and advanced users, it is common practice to save the image of the nodule and determine the correct interpretation later by reviewing the image and analyzing its features [12]. In a clinical setting, finalizing the report with an appropriate recommendation on how to proceed with the finding is crucial for sustainable use of medical capacities.

The latest advancements in medical imaging, the field of artificial intelligence (AI), could support clinicians in the interpretation of imaging characteristics of nodules. In particular, the development of technologies such as the advanced language model ChatGPT 4.0 have profoundly affected our daily lives, and more recently, the broader scientific community. These AI models are capable of understanding and generating human-like text, not only transforming the way we interact with information technology but also beginning to permeate various sectors, including healthcare [13]. This integration of AI into medical practices offers promising avenues for enhancing diagnostic workflows, personalizing patient care, and facilitating the analysis of vast datasets, which is instrumental in advancing medical research and knowledge [14,15]. Also, it is increasingly being recognized as a transformative tool in the education and training of medical students [16].

As a test for the potential use of ChatGPT 4.0 in interpreting ultrasound findings, application areas with clearly defined assessment criteria that clinicians can follow are particularly suitable. Since thyroid findings, unlike for example cervical lymph nodes, can be interpreted relatively well using criteria such as ACR-TIRADS 2017, there are already several studies investigating the application of AI in the context of the thyroid. For instance, Li et al. (2023) and Namsena et al. (2024) proposed an AI system for interpreting ultrasound images of thyroid nodules, which resulted in increased diagnostic accuracy [17,18] while Guo et al., 2024 compared how ChatGPT 4.0 performed in answering patient questions about the thyroid compared to surgeons and found that the responses provided by the large language model were more satisfactory to patients than those of experts, regardless of their level of training [19].

In the era of rapidly evolving medical imaging and artificial intelligence, our study investigates the potential of integrating conventional thyroid ultrasound (US) diagnostics with AI-driven analysis. We focus on the application of the ACR-TI-RADS 2017 guidelines—a widely accepted standard for assessing thyroid nodules—to establish a robust framework for risk stratification.

Specifically, our objectives are to (i) evaluate whether ChatGPT 4.0 can effectively support both experienced and inexperienced US users in interpreting thyroid US reports and (ii) to determine if ChatGPT 4.0 can accurately apply the ACR-TI-RADS 2017 criteria to distinguish between nodules that require immediate fine needle aspiration (FNA) and those that need follow-up examinations.

Through this dual-pronged approach, the study aims to clarify the role of AI as a complementary tool in thyroid diagnostics, highlighting both its potential benefits and current limitations.

## 2. Materials and Methods

### 2.1. ACR-TIRADS Criteria of 2017 and US Reports

**Figure 1:**
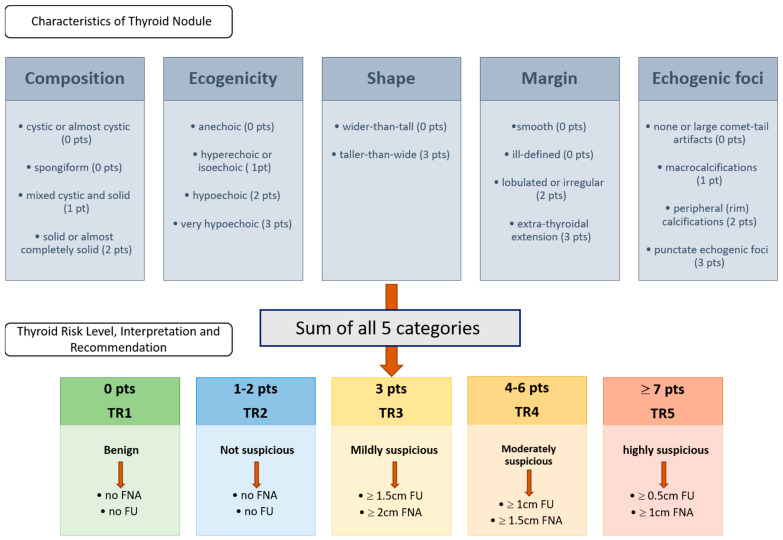
ACR TI-RADS 2017 classification according to ACR White Paper 2017 [8]. The upper row displays the five characteristics that must be evaluated according to the ACR-TI-RADS classification system. The rater assigns the corresponding points to the characteristics of the thyroid nodule, sums up the points, and proceeds based on the resulting thyroid risk level, interpretation, and recommendation, which are shown in the second row. Abbreviations: pt = point; pts = points; TR = thyroid risk.

The fictitious US reports were created by an otolaryngologist (EFSUMB Level II, Head and Neck) based on the ACR-TI-RADS 2017 criteria. The classification [8] assigns points based on various US features of the nodules, categorizing them into risk levels that suggest the likelihood of malignancy. The main components of the ACR-TI-RADS 2017 classification include composition, echogenicity, shape, margin, and echogenic foci. Based on the total points, nodules are categorized into TI-RADS levels. Each category is associated with recommendations for follow-up or biopsy, with higher TI-RADS categories indicating a greater likelihood of malignancy and a stronger recommendation for biopsy: The classification is shown in Figure 1.

**Figure 2:**
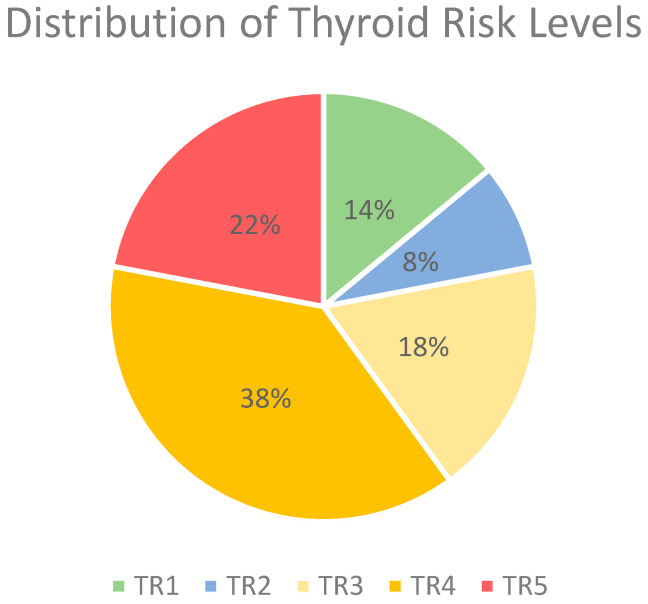
Distribution of thyroid risk levels in %. Abbreviations: TR, thyroid risk.

Due to the substantial data protection requirements involved in testing the use of AI for analyzing written US findings—an application that has not yet been clinically established or approved—the research group decided to conduct this initial investigation using fictional reports based on structured US reports used in daily routine. For the creation of findings, the generalized ACR-TI-RADS 2017 criteria were adhered to, and the individual elements were objectively described. The reports were designed to cover a wide spectrum of thyroid nodule characteristics representative of real-world clinical scenarios. Each report included detailed descriptions of nodule shape (size in cm, transverse and anterior-posterior dimension), composition, echogenicity, margins, presence of echogenic foci, and other features relevant to ACR-TI-RADS 2017 categorization [8]. To focus on whether the large language model can detect and accurately interpret suspicious or potentially malignant descriptions, two-thirds of the reports included characteristics of nodules corresponding to a TI-RADS level of 4 or 5. Because fictitious US reports were used and no patient data were included, approval from the local ethics committee was not necessary. The distribution of TR-Levels can be seen in Figure 2. Each report rating was double-checked by the creator by using the online ACR-TIRADS-2017 calculator (https://tiradscalculator.com/, accessed on 2–5 February 2024).

### 2.2. Rating

These fictional findings were evaluated in consensus by a team of experienced US users. The expert team consisted of a radiologist, an endocrinologist, and an otolaryngologist, each with at least three years of expertise in thyroid ultrasound. All outcomes were independently adjudicated by two of the authors (M.H. Lerchbaumer and L. Kozarzewski), and disagreements were resolved by a third author (S. Dommerich) during an in-person meeting held within one day for comparison to ChatGPT 4.0 and the inexperienced user as a gold standard. US reports were also interpreted by an inexperienced user (medical student in the 3rd year of education) within one day. Both the experts and the inexperienced US users were allowed to use the ACR-TI-RADS 2017 guidelines/template to assign a risk level category (TR1 to TR5) to each report and to recommend a consecutive procedure such as FNA, follow-up, or no monitoring at all [8].

### 2.3. ChatGPT 4.0 Intervention

For the AI intervention, we used ChatGPT 4.0, which is accessible under a paid model. It is a more sophisticated version of OpenAI Inc.’s language model than its freely accessible predecessor, 3.5, offering improvements in understanding context, generating human-like responses, and handling complex instructions. Compared to version 3.5, it showcases better performance in nuanced language understanding, reduced biases, and a broader knowledge base, allowing for more accurate and contextually appropriate interactions across diverse subjects and languages [20].

ChatGPT 4.0 was tasked with evaluation of the same set of 100 US reports. For this purpose, the model was provided with the text of each report and instructed to rate the thyroid nodules according to the ACR-TI-RADS 2017 criteria with the following command: “*Rate this report of an US examination of the thyroid gland according to the ACR-TI-RADS (2017) and give me the cumulated score and the TI-RADS Level and tell me if a fine needle aspiration or follow up is recommended*”. (prompt. ChatGPT 4.0, 12, 13, 14 February and 7 March 2024). To check the intrarater-reliability, all US reports were entered into ChatGPT 4.0 in random order for analysis (prompt. 13–14 March 2024) following the same command. No additional contextual information or training specific to the task was given to ChatGPT beyond its pre-existing knowledge and capabilities up to its last update for this analysis.

During the second round, the US reports were analyzed again by ChatGPT 4.0 using a more specific command by adding pre-existing knowledge:

“*rate the following US reports according to these criteria:*

*The ACR TI-RADS 2017 classification assigns points based on various US features of the nodules, categorizing them into risk levels that suggest the likelihood of malignancy. The main components of the ACR TI-RADS classification include:*

*1.*  *Composition*
  - *Cystic or almost completely cystic (0 points)*
  - *Spongiform (0 points)*
  - *Mixed cystic and solid (1 point)*
  - *Solid or almost completely solid (2 points)*
*2.*  *Echogenicity*
  - *Anechoic (0 points)*
  - *Hyperechoic or isoechoic (1 point)*
  - *Hypoechoic (2 points)*
  - *Very hypoechoic (3 points)*
*3.*  *Shape*
  - *Wider-than-tall (0 points)*
  - *Taller-than-wide (3 points)*
*4.*  *Margin*
  - *Smooth (0 points)*
  - *Ill-defined (0 points)*
  - *Lobulated or irregular (2 points)*
  - *Extra-thyroidal extension (3 points)*
*5.*  *Echogenic Foci*
  - *None or large comet-tail artifacts (0 points)*
  - *Macrocalcifications (1 point)*
  - *Peripheral (rim) calcifications (2 points)*
  - *Punctate echogenic foci (3 points)*

*Based on the total points, nodules are categorized into TI-RADS (TR) levels. Each category is associated with recommendations for follow-up or biopsy, with higher TI-RADS categories indicating a greater likelihood of malignancy and a stronger recommendation for biopsy:*

*TR1: Benign (0 points)*
*TR2: Not suspicious (2 points or less)*
*TR3: Mildly suspicious (3 points)*
  - *bigger than or exactly 1.5 cm follow-up*
  - *bigger than or exactly 2.5 cm FNA*
*TR4: Moderately suspicious (4–6 points)*
  - *bigger than or exactly 1.0 cm follow-up*
  - *bigger than or exactly 1.5 cm FNA*
*TR5: Highly suspicious (7 points or more)*
  - *bigger than or exactly 0.5 cm follow-up*
  - *bigger than or exactly 1.0 cm FNA*

*analyze the reports according to composition, echogenicity, shape (given the fact that size is stated in transverse x anterior-posterior dimension) and tell if the node is taller than wide, rate the margins and echogenic foci and state the cumulated score and the TR-Level and tell me if a FNA or follow-up is necessary*”.

**Figure 3:**
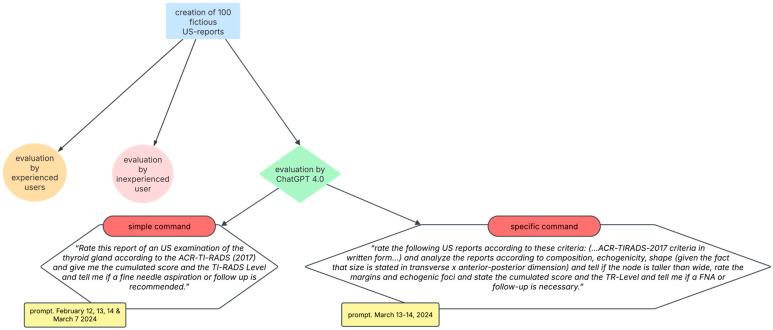
Summary of methodology. Abbreviations: US, ultrasound; TR, thyroid risk; FNA, fine needle aspiration.

A summary of the methodology is shown in Figure 3.

### 2.4. Data Collection and Analysis

The ACR-TI-RADS 2017 categories assigned by ChatGPT were recorded and compared with the consensus classification assigned by the experienced group. The primary outcome measure was the accuracy of ChatGPT’s ratings, assessed by calculating the percentage of reports for which ChatGPT’s assigned category matched the experts. Secondary analyses included evaluating the consistency of ChatGPT’s ratings across different nodule characteristics, identifying specific features that may have influenced the accuracy of its assessments, and comparing the diagnostic accuracy of the AI language model’s ratings to those of an inexperienced US user.

### 2.5. Statistical Analysis

Descriptive statistics were used to summarize the characteristics of the US reports and the distribution of ACR-TI-RADS 2017 categories assigned by ChatGPT 4.0, the expert team, and the inexperienced user. The intrarater agreement between two ChatGPT 4.0 ratings as well as the interrater agreement between the expert consensus and ChatGPT 4.0 and the expert consensus and the inexperienced US user’s rating were evaluated using Cohen’s kappa coefficient. A kappa value of 0 indicated no agreement beyond chance, while a value of 1 indicated perfect agreement. Values under 0.40 were considered to reflect poor agreement, 0.40–0.59 moderate agreement, 0.60–0.79 substantial agreement, and 0.80–1.00 almost perfect agreement [21]. A *p*-value of <0.05 was considered statistically significant.

The diagnostic performance of the two analyses conducted by ChatGPT 4.0, alongside the inexperienced US user’s evaluation of thyroid nodules, was assessed through the calculation of sensitivity, specificity, positive predictive value (PPV), and negative predictive value (NPV) utilizing cross-tabulation methods with the expert group’s rating serving as reference. A two-sided significance threshold of α = 0.05 was established to denote statistical significance. Pearson’s chi-square test was employed to examine associations between categorical variables. The diagnostic accuracy of the ratings provided by ChatGPT and the student was calculated using the expert consensus as the comparative gold standard. This means that the decisions or classifications made by the expert group were considered the true, or reference, values against which the performance of ChatGPT and the student was measured.

All statistical analyses were performed using statistical software (SPSS, version 26, IBM Corp., Armonk, NY, USA).

## 3. Results

### 3.1. Intrarater Reliability of ChatGPT 4.0

The intrarater reliability analysis of two staggered ChatGPT 4.0 evaluations of thyroid US reports revealed varying degrees of agreement across different categories, as quantified by Cohen’s kappa values. The ACR-TI-RADS-Score (calculated as the total number of points of each category) displayed a Cohen’s kappa of 0.380, indicating poor agreement, with a statistically significant *p*-value of <0.001. The TI-RADS category (TR) showed a moderate level of agreement with a kappa of 0.509, also with a *p*-value of <0.001. The decision for FNA yielded a substantial agreement with a kappa of 0.653, and the recommendation for follow-up had a moderate agreement with a kappa of 0.588, both statistically significant with *p*-values of <0.001.

When analyzing specific US characteristics, the agreement for composition and echogenicity was rated as moderate (kappa = 0.413) and substantial (kappa = 0.677), respectively, both with *p*-values < 0.001. Shape analysis could not be evaluated due to unspecified reasons. Margins and echogenic foci showed substantial agreement with kappa values of 0.685 and 0.907, respectively, suggesting a high level of consistency in these evaluations, with *p*-values < 0.001 indicating statistical significance.

These findings indicate a varied level of consistency in the repeated analysis of thyroid US reports by ChatGPT 4.0, with the strongest agreement observed in the evaluation of echogenic foci.

No Cohen’s kappa could be calculated for “shape” because ChatGPT 4.0 did not correctly identify any “taller-than-wide” signs. Consequently, all 100 reports were rated with “0” for shape and did not show any variations. This made it mathematically impossible to calculate Cohen’s kappa, because it relies on the presence of variation in the data sets of ratings, to compute how much the observed agreement exceeds what would be expected by chance alone.

For further details see Table 1.

### 3.2. Interrater Reliability Between Group of Experts and ChatGPT 4.0

Since the results of the intrarater reliability differ from each other, the interrater reliability between the expert group and ChatGPT 4.0 was conducted for both ChatGPT 4.0 analyses (referred to as “analysis I” and analysis “II”). The results demonstrate a range of agreement from moderate to almost perfect, based on Cohen’s kappa coefficient. Specifically, the ACR-TI-RADS-score agreement was moderate for both analyses I and II (kappa = 0.474 and 0.492, respectively), with accuracy rates of 53% and 54%. For the TI-RADS category (TR), a moderate agreement was observed in both analyses, with kappa values of 0.499 and 0.402 and accuracy rates of 60% and 51%, respectively.

When considering the recommendations for FNAFNA, analysis I showed a moderate agreement (kappa = 0.573) with an accuracy of 81%, whereas analysis II demonstrated a moderate agreement (kappa = 0.444) with a 74% accuracy. The recommendation for follow-up exhibited a moderate agreement in analysis I (kappa = 0.400) and a poor agreement in analysis II (kappa = 0.334), with accuracy rates of 71% and 67%, respectively.

The composition feature in analysis II achieved an almost perfect agreement (kappa = 0.933) with a 97% accuracy, significantly higher than analysis I (kappa = 0.440, 66% accuracy). Echogenicity showed almost perfect agreement in both analyses, with exceptionally high accuracy (97% in analysis I and 78% in analysis II). Margins and echogenic foci also displayed high levels of agreement, particularly in analysis I (kappa = 0.922 and 0.880, respectively), indicating nearly perfect concordance, with accuracies over 95%. For further details see Table 2.

### 3.3. Interrater Reliability Between Group of Experts and Medical Student

The results of the interrater reliability between the analyses of thyroid US exams by the group of experts and the medical student are presented in Table 3. These findings demonstrate an almost perfect agreement across all evaluated parameters, with Cohen’s kappa values ranging from 0.909 for the ACR-Score to 1.0 for margins, indicating a highly consistent and precise level of agreement between the two raters. The *p*-values for all categories were less than 0.001, further supporting the statistical significance of this high level of agreement. Accuracy percentages also reflected this strong concordance, with the lowest accuracy being 92% for the ACR-Score and the highest being 100% for margins. Other categories such as TR (thyroid risk), FNA (fine needle aspiration), follow-up recommendations, composition, echogenicity, shape, and echogenic foci, also showcased high agreement with accuracy percentages ranging from 95% to 99%.

### 3.4. Diagnostic Performance of ChatGPT 4.0 and Inexperienced US User

For the determination of FNA recommendation, ChatGPT-I exhibited a sensitivity of 61%, specificity of 94%, PPV of 85%, and NPV of 79%, with a significant chi-square value (χ2 (1) = 34.952, *p* < 0.001). ChatGPT-II showed slightly lower performance with a sensitivity of 55%, specificity of 87%, PPV of 72%, NPV of 76%, and a chi-square value of χ2 (1) = 20.532, *p* < 0.001. In stark contrast, the student’s analysis demonstrated markedly higher diagnostic performance with a sensitivity of 97%, specificity of 98%, PPV of 97%, NPV of 98%, and a chi-square value of χ2 (1) = 91.691, *p* < 0.001.

Regarding the recommendation for a follow-up examination, ChatGPT-I achieved a sensitivity of 96%, specificity of 43%, PPV of 65%, and NPV of 91%, with a chi-square value of χ2 (1) = 21.830, *p* < 0.001. ChatGPT-II reported a sensitivity of 98%, specificity of 34%, PPV of 63%, NPV of 94%, and a chi-square value of χ2 (1) = 18.254, *p* < 0.001. The student’s performance remained consistently high with a sensitivity of 98%, specificity of 96%, PPV of 96%, NPV of 98%, and a chi-square value of χ2 (1) = 88.341, *p* < 0.001. For further details see Table 4.

### 3.5. Chat GPT 4.0’s Analysis of US Report with Explicit Instruction Containing the Description of the ACR-TI-RADS Criteria of 2017

ChatGPT 4.0 could not process the explicit command describing the ACR-TI-RADS criteria of 2017 as stated in the “Methods” section. After entering the command, ChatGPT 4.0 answered “It seems there was an issue processing your request due to its complexity and limitations on executing code for such tasks. If you need detailed analysis according to the ACR TI-RADS 2017 criteria for each US report, it’s recommended to manually apply the criteria based on the described characteristics of the nodules, as the automated system encountered a limitation in processing the complex logic required for this specific task”. (prompt. ChatGPT 4.0, 13 and 14 March 2024).

## 4. Discussion

The integration of AI into the medical field has opened new horizons for enhancing diagnostic accuracy and streamlining healthcare services. Our study aimed to evaluate the efficacy of ChatGPT 4.0 in interpreting thyroid US reports according to the ACR-TI-RADS 2017 criteria and compare its performance to medical experts and an inexperienced US user. The analysis revealed moderate to almost perfect agreement in different aspects of the US reports but highlighted inconsistencies in reliability and performance. While ChatGPT 4.0 demonstrated substantial agreement in assessing echogenic foci, its ability to evaluate other nodule characteristics, such as composition and margins, was less consistent. Interrater reliability between ChatGPT and experts varied across different aspects of the evaluation, indicating that AI can provide useful insights but should function as a complementary tool rather than a replacement for human expertise.

In the evaluation of 100 thyroid US report datasets, the first analysis by ChatGPT was conducted over different days, revealing variability in the application of ACR-TI-RADS 2017 criteria. For example, ChatGPT omitted the evaluation of nodule composition during one session and misclassified nodules based on incorrect ACR scores. In a subsequent analysis conducted consecutively on the same day, ratings again changed, leading to variable intrarater reliability. This reflects ChatGPT 4.0’s inconsistency in repeated analyses, as well as challenges in achieving consistent performance despite unchanged input commands since there was no definitive improvement in ChatGPT’s interpretations over time.

The significantly varying intrarater reliability of ChatGPT 4.0 reflects the persistent changes in criteria shown during the analysis in the output of results, despite the input command remaining unchanged. This inconsistency indicates that even when presented with the same information or task, ChatGPT 4.0’s evaluations or interpretations may fluctuate over time. This variability could be due to the model’s processing or the application of different internal criteria for similar tasks, highlighting a challenge in achieving consistent performance across repeated tasks or analyses [22]. The interrater reliability between the expert group and ChatGPT 4.0 varied across analyses, with moderate to almost perfect agreement in different aspects of the US reports.

One major factor influencing ChatGPT’s performance is the complexity and ambiguity of the task. The model performs better in structured, objective tasks such as echogenicity (κ = 0.953, 0.68) and echogenic foci (κ = 0.880, 0.911), where the classification criteria are well-defined and widely available in training data. However, it struggles with more subjective and complex classifications, such as thyroid risk (TR) (κ = 0.499, 0.402) and follow-up recommendations (κ = 0.400, 0.334), which require clinical judgment and nuanced interpretation beyond simple pattern recognition.

Another contributing factor is ChatGPT’s reliance on training data and potential knowledge gaps. While the model is trained on the publicly available medical literature and guidelines, it lacks direct exposure to expert-level decision-making and proprietary clinical datasets used by radiologists. This limitation may explain why ChatGPT has difficulty applying the American College of Radiology (ACR) scoring system and thyroid risk classification [8], as these tasks require a deeper contextual understanding of radiological features. Additionally, ChatGPT performs better in binary classifications compared to multi-class classifications. For example, in echogenic foci and echogenicity, where the classification options are relatively limited, ChatGPT achieves high interrater reliability (κ > 0.88, almost perfect agreement). However, its performance drops in thyroid risk stratification (TR) and follow-up recommendations, where the classification is more complex and requires making finer distinctions. This suggests that ChatGPT may struggle with more detailed, multi-tiered decision-making compared to simpler yes/no classifications.

An additional notable factor is model variability between runs. The differences observed between ChatGPT-I and ChatGPT-II, particularly in TR classification, FNA recommendations, and follow-up assessments, indicate that ChatGPT does not always provide consistent results for the same input. Since language models generate responses probabilistically, even minor variations in prompts can lead to different outputs, which can reduce reliability in clinical settings where consistency is critical.

Furthermore, it is important to consider the threshold for clinical acceptability. While ChatGPT achieves high accuracy (97%) in echogenicity and margins, it demonstrates only moderate agreement in FNA recommendations (κ = 0.573, 0.444). Given that FNA decisions directly impact patient management, inconsistent results could lead to unnecessary procedures or missed malignancies, raising concerns about the model’s clinical applicability.

The overall moderate agreement in all categories but composition indicates that while ChatGPT 4.0 can provide valuable insights, it should complement rather than replace human expertise in thyroid nodule evaluation. This aligns with recommendations in the current literature that users, particularly in the medical field, should verify AI-generated information with trustworthy sources [23,24].

When compared to an inexperienced US user, ChatGPT 4.0’s performance was less robust. The inexperienced user achieved almost perfect agreement with the expert group, highlighting the effectiveness of traditional medical education in imparting the knowledge and skills necessary for accurate US interpretation [11,25].

In terms of recommending appropriate treatments, the inexperienced user outperformed ChatGPT, particularly in sensitivity and specificity when recommending follow-up examinations and FNAs. Examining the diagnostic performance, it is clear that one of the main limitations of ChatGPT models is their lower sensitivity in the FNA setting, meaning they may fail to detect true cases of disease. This makes them unreliable for primary diagnosis. Additionally, their poor specificity in the follow-up setting suggests they classify too many healthy individuals as needing follow-up, leading to unnecessary procedures and increased patient anxiety. While ChatGPT models perform relatively well in terms of specificity in FNA and NPV in follow-up, their inconsistent performance across different metrics makes them less reliable compared to an inexperienced user.

Sensitivity, or the true positive rate, measures how well a model identifies patients with malignant nodes. In the FNA setting, ChatGPT-I (61%) and ChatGPT-II (55%) demonstrate relatively low sensitivity, indicating that they miss a significant number of true positive cases, potentially leading to underdiagnosis. In contrast, the inexperienced user achieved a much higher sensitivity (97%), ensuring more accurate detection of malignant nodules. However, in the follow-up setting, all models exhibit high sensitivity, with ChatGPT-I at 96%, ChatGPT-II at 98%, and the inexperienced user at 98%. This suggests that while ChatGPT performs well in identifying cases requiring further monitoring, its ability to detect malignancies in the FNA setting remains limited.

Specificity, or the true negative rate, evaluates how well the models correctly identify patients without the condition and therefore benign nodes. In the FNA scenario, ChatGPT-I (94%) and ChatGPT-II (87%) show strong specificity, effectively identifying healthy cases. The inexperienced user performed even better, achieving a specificity of 98%. However, in the follow-up setting, ChatGPT models perform poorly, with ChatGPT-I at 43% and ChatGPT-II at 34%. This indicates that these models frequently classify healthy individuals as needing follow-up, potentially leading to unnecessary medical interventions. The inexperienced user, with a specificity of 96%, demonstrated a much better balance between sensitivity and specificity.

Positive predictive value (PPV) represents the likelihood that a positive test result accurately indicates disease. In the FNA setting, ChatGPT-I (85%) and ChatGPT-II (72%) demonstrate moderate PPV, suggesting that while their positive classifications are reliable, they are not perfect. The inexperienced user, with a PPV of 97%, provided significantly greater confidence in positive diagnoses. In the follow-up setting, PPV is lower for all models, with ChatGPT-I at 65%, ChatGPT-II at 63%, and the inexperienced user at 96%. This suggests that ChatGPT models generate more false positives in follow-up recommendations, increasing the likelihood of unnecessary tests and treatments.

Negative predictive value (NPV) measures how accurately a negative result indicates the absence of malignancy. In the FNA setting, ChatGPT-I (79%) and ChatGPT-II (76%) have lower NPV compared to the inexperienced user (98%), indicating that a negative result from ChatGPT is less reliable and may still overlook true positive cases. However, in the follow-up setting, all models perform well, with ChatGPT-I at 91%, ChatGPT-II at 94%, and the inexperienced user at 98%. This suggests that when ChatGPT classifies a patient as not needing follow-up, its prediction is generally reliable.

In summary, while ChatGPT 4.0 provided comprehensible explanations for patient education, it failed to match the diagnostic accuracy of a beginner-level human evaluator. This finding suggests that while AI technologies like ChatGPT 4.0 offer innovative tools for learning and decision support, the foundational understanding and critical thinking developed through conventional training remain indispensable in clinical practice [14,26]. While ChatGPT appears capable of generating straightforward and comprehensible explanations of medical conditions for patient understanding, it cannot replace the expertise of a healthcare professional [27,28]. The study findings indicate that the inexperienced user outperformed ChatGPT 4.0, achieving almost perfect agreement with experts across all evaluated parameters. This may suggest that human cognitive reasoning, pattern recognition, and familiarity with clinical workflows play a crucial role in ensuring reliable decision-making, whereas AI relies on statistical pattern analysis, which may lack the depth of understanding required for nuanced medical assessments [29,30].

We were surprised to find that the interpretation of findings by ChatGPT 4.0 did not significantly differ between commands with explicit instructions, including a detailed breakdown of all evaluation criteria, and those using simple commands. Despite explicitly formulating a command to include size evaluation, ChatGPT 4.0 was unable to interpret complex criteria such as the “taller-than-wide” sign, assigning “0” points for shape when 3 points should have been given for suspicious nodules. These errors affected the cumulative ACR-TI-RADS score, the TR-Level, and subsequent treatment recommendations. Because ChatGPT 4.0 was unable to consider the taller-than-wide sign and thus the shape of the nodules at all, suspicious findings are mistakenly rated as too benign (i.e., too healthy). This can lead to undertreatment and pose a potential risk due to the lack of response to suspicious findings, potentially resulting in the oversight of malignant cases [31].

The interpretation of shape appears to be challenging for interpreters without a medical background. Trimboli et al. (2024) compared a computer scientist, representing a non-medical individual, with two medical experts in interpreting ultrasound reports of thyroid nodules according to the ACR-TI-RADS and the Korean and European classification systems. While the computer scientist performed well with the ACR-TI-RADS system, he struggled with the more pattern-based Korean and European classification systems. Notably, the only category in the ACR-TI-RADS system where the computer scientist’s results deviated from those of the experts was the shape of the nodules, which he was unable to interpret accurately without medical knowledge [32]. This limitation is also reflected in our ChatGPT analysis and might suggest that accurate interpretation of nodule shape requires medical expertise—an understanding that neither the computer scientist nor the large language model possesses.

Xia et al. (2025) tested the free predecessor version, ChatGPT 3.5, in interpreting thyroid ultrasound findings and reached similar conclusions; the large language model failed to satisfactorily answer questions regarding FNA (fine needle aspiration) or further therapeutic approaches. The authors attributed this limitation to the abundance of publications advocating for various interventional procedures, such as surgical resection or thermal ablation [33]. Nonetheless, the model should at least be capable of addressing the necessity of FNA, especially given the availability of clearly established ACR-TIRADS criteria for this question. These criteria were even included verbatim in the command provided for execution.

Additionally, ChatGPT’s inability to maintain consistent accuracy in applying ACR criteria underscores the limitations of current AI models in grasping nuanced or subjective diagnostic criteria. Its lack of understanding of explicit guidelines and tendency to generate “artificial hallucinations” further emphasize the need for improved training and refinement. These observations highlight the challenges and limitations associated with employing AI models like ChatGPT for the nuanced task of medical image classification and reporting [34]. The variability in performance across different sessions suggests that while AI has the potential to augment the diagnostic process, reliability and consistency remain critical areas for improvement. It also underscores the importance of continuous monitoring and adjustment of AI systems to ensure alignment with clinical guidelines and standards [35]. This is underlined by the fact that the language model could not process the explicit instructions on how to analyze the nodes. The analysis underscored ChatGPT 4.0’s limitations in processing highly specialized and explicit medical instructions, particularly those requiring the interpretation of complex clinical guidelines. Certain limitations of ChatGPT stem from its foundational data model. Notably, it is not informed about events post-September 2021 and does not possess the ability to learn from its interactions. Furthermore, as highlighted by its developers, ChatGPT might generate what are known as “artificial hallucinations”—content that may be nonsensical or inaccurately related to specific sources—a challenge that becomes increasingly difficult to identify given the model’s advancing realism [22,36]. While large language models (LLMs) can generate coherent and contextually relevant responses, they lack a true understanding of underlying medical concepts. Their outputs are based on statistical patterns learned from training data, which may not fully capture the complexities of medical diagnoses. In cases where deep contextual comprehension is essential, such as rare diseases or complex patient scenarios, relying solely on LLM-generated responses could result in inaccurate or incomplete information [37].

Furthermore, the black-box nature of LLMs poses a challenge, as they lack explainability and transparency in their reasoning. The model does not provide clear justifications for its decisions, making it difficult for healthcare professionals to trust and validate its recommendations. This lack of interpretability limits the model’s usefulness in critical decision-making situations, where clinicians need a well-defined rationale behind suggested diagnoses or treatment options. LLMs are trained on vast datasets that may contain inherent biases or inaccuracies, which can be reflected in their outputs. Even when given explicit prompts designed to counteract bias or errors, the model may still produce responses influenced by the biases present in its training data. Since LLMs generate text based on learned statistical patterns rather than true understanding, they can inadvertently reinforce and propagate existing biases or misinformation. This limitation makes it essential to carefully monitor and mitigate biases in both the training process and real-world applications, especially in fields like medicine, where accuracy and fairness are critical [37,38].

To enhance the consistency of AI models in diagnostic workflows and reduce variability between raters, several key strategies should be implemented.

One crucial step is refining AI training by incorporating expert-labeled data. AI models should be trained on large, well-annotated datasets created with input from experienced radiologists and endocrinologists to improve the accuracy and reliability of decision-making. Additionally, supervised learning techniques, such as reinforcement learning from expert feedback (RLHF), could help align AI-generated classifications with expert-level reasoning, ensuring that the model learns from real-world clinical expertise.

Another important approach is improving AI decision-making standardization. Instead of relying solely on probabilistic text generation, AI models should integrate structured decision pathways that strictly adhere to ACR-TI-RADS criteria. This would ensure more consistent and reproducible assessments, minimizing variability in classification. Furthermore, implementing rule-based models alongside AI predictions could help reduce inconsistencies, particularly in complex or borderline cases where AI responses may vary between analyses.

Beyond statistical validation, it is also essential to establish clinical acceptability thresholds for AI performance. While statistical significance provides useful insights, clinical relevance should be the primary factor when evaluating AI-assisted diagnostic tools. Establishing clear performance benchmarks would help determine whether AI models can be safely implemented in real-world diagnostic workflows.

A more collaborative approach between AI and human experts may also improve reliability. Rather than using ChatGPT as an autonomous decision-maker, AI models should function as decision-support tools, generating suggested risk scores while leaving final classification and treatment decisions to clinicians. A hybrid approach, where AI-generated classifications are reviewed and modified by trained professionals, could enhance diagnostic efficiency without compromising accuracy.

One of the biggest challenges in AI adoption is the lack of interpretability in AI-generated decisions. To build trust among healthcare professionals, AI models should provide greater transparency in their reasoning. For example, rather than simply outputting a classification, the AI should clearly highlight the specific features that influenced its rating (e.g., “hypoechoic with irregular margins = TR4”). This would allow clinicians to understand and validate AI-driven recommendations, making the technology more practical for clinical use. By implementing these strategies, AI models can become more consistent, interpretable, and clinically useful, ultimately improving their integration into real-world diagnostic workflows [30].

A comparable study was conducted [39], which shows similar results. It examines the capabilities, limitations, and potential of ChatGPT 4.0 as an advanced AI language model, in interpreting thyroid US reports, providing diagnoses, and suggesting treatment plans. Unlike our study, they were able to use 109 real thyroid cancer cases from diverse clinical settings. The study compares ChatGPT 4.0’s performance with doctors of varying expertise and evaluates the model’s consistency, interpretability, and human-like qualities through a Turing Test.

It excelled in structuring reports, employing professional terminology, and maintaining clarity of expression, achieving high ratings in these areas. However, its diagnostic accuracy was moderate and notably lower compared to human doctors. Reproducibility experiments further highlighted variability in its diagnostic accuracy, underlining the need for improved consistency in repeated tasks, which matches our results. The Turing Test revealed that ChatGPT 4.0-generated reports were often indistinguishable from human-written ones, with many being rated as “likely human-written”. To enhance transparency and trust in its diagnostic process, the Chain of Thought method was applied, breaking down the AI’s decision-making steps for better interpretability.

Doctors provided mixed feedback on Chat GPT 4.0’s performance. While its reports were acknowledged for being detailed and clear, concerns were raised about inaccuracies and overly complex sections, pointing to areas for improvement. To address these challenges, the study group introduced “ThyroAIGuide”, an online diagnostic platform that automates report generation, provides risk assessments, and offers treatment recommendations [39].

While AI, and particularly large language models, increasingly become a part of our daily lives, it is crucial to identify how these tools can be utilized to simplify workflows and processes in medicine. At the same time, it remains a challenge to discern the fine line that separates true information from information presented as truth. Especially in clinical practice, where physicians are burdened with growing organizational and bureaucratic tasks, the proper application of AI can alleviate significant workloads and save time by enabling quick copying and pasting of findings followed by immediate interpretation [40].

However, our study results indicate that the information and perceived assistance offered by such tools should never be accepted uncritically but must always undergo careful evaluation. The risks associated with this are reflected in our findings. The inability of ChatGPT 4.0 to filter information regarding nodule shape leads to down-staging and, consequently, an underestimation of potentially malignant and high-risk findings that require monitoring. Our examinations using realistic, fictional case reports demonstrate that despite the hype surrounding AI, it should not be trusted without scrutiny. Finally, it should be added that a limitation of this study is its reliance on simulated ultrasound reports modeled after the ACR-TI-RADS 2017 standard, which may constrain the generalizability of the findings to real-world clinical settings due to the absence of actual patient data. However, the use of fictional reports was necessary to comply with stringent data protection requirements.

## 5. Conclusions

While this study highlights the potential of AI tools like ChatGPT 4.0 to support clinicians in evaluating thyroid US reports, it also emphasizes critical limitations, particularly regarding consistency, diagnostic accuracy, and the ability to process complex medical criteria. ChatGPT’s performance falls notably short compared to human evaluators, especially when measured against even a beginner’s level of expertise. Future research should prioritize improving AI models’ accuracy, reliability, and interpretability through targeted training and algorithm enhancements. Despite the promise of AI, traditional medical education remains essential, enabling clinicians to identify and correct AI-driven errors and ensuring the safe and effective integration of AI into clinical practice.

## Figures and Tables

**Table 1:** Results of statistical measure of intrarater reliability of ChatGPT 4.0. Abbreviations: ACR, American College of Radiology; TR, thyroid risk; FNA, fine needle aspiration.

	Cohen’s Kappa	Strength of Agreement	*p*-Value
ACR-TI-RADS-Score	0.380	Poor	<0.001
TR	0.509	Moderate	<0.001
FNA	0.653	Substantial	<0.001
Follow-Up	0.588	Moderate	<0.001
Composition	0.413	Moderate	<0.001
Echogenicity	0.677	Substantial	<0.001
Shape	-	-	-
Margins	0.685	Substantial	<0.001
Echogenic foci	0.907	Almost perfect	<0.001

**Table 2:** Results of statistical measure of interrater reliability between experts’ consensus and the first (I) and second (II) ChatGPT 4.0 rating. Abbreviations: ACR, American College of Radiology; TR, thyroid risk; FNA, fine needle aspiration.

	Cohen’s Kappa		Strength of Agreement		*p*-Value	Accuracy	
	I	II	I	II	I and II	I	II
ACR-Score	0.474	0.492	Moderate	Moderate	<0.001	53%	54%
TR	0.499	0.402	Moderate	Moderate	<0.001	60%	51%
FNA	0.573	0.444	Moderate	Moderate	<0.001	81%	74%
Follow-Up	0.400	0.334	Moderate	Poor	<0.001	71%	67%
Composition	0.440	0.933	Moderate	Almost perfect	<0.001	66%	97%
Echogenicity	0.953	0.68	Almost perfect	Almost perfect	<0.001	97%	78%
Shape	-	-	-	-	-	79%	79%
Margins	0.922	0.665	Almost perfect	Substantial	<0.001	97%	86%
Echogenic foci	0.880	0.911	Almost perfect	Almost perfect	<0.001	96%	96%

**Table 3:** Results of statistical measure of interrater reliability between the expert’s and the inexperienced user’s rating. Abbreviations: ACR, American College of Radiology; TR, thyroid risk; FNA, fine needle aspiration.

	Cohen’s Kappa κ	Strength of Agreement	*p*-Value	Accuracy
ACR-Score	0.909	Almost perfect	<0.001	92%
TR	0.933	Almost perfect	<0.001	95%
FNA	0.958	Almost perfect	<0.001	98%
Follow-Up	0.940	Almost perfect	<0.001	97%
Composition	0.933	Almost perfect	<0.001	97%
Echogenicity	0.922	Almost perfect	<0.001	95%
Shape	0.970	Almost perfect	<0.001	99%
Margins	1	Almost perfect	<0.001	100%
Echogenic foci	0.971	Almost perfect	<0.001	99%

**Table 4:** Diagnostic performance in the determination of indication of fine needle aspiration or follow-up examination of the ChatGPT 4.0 analyses and the student’s analysis. Sensitivity, specificity, PPV, and NPV are given in percentages with corresponding *p*-values and χ2 (degrees of freedom stated in brackets). Abbreviations: FNA, fine needle aspiration; PPV, positive predictive value; NPV, negative predictive value; χ2, Pearson’s chi-square test; ChatGPT-I, ChatGPT 4.0 analysis I; ChatGPT-II, ChatGPT 4.0 analysis II; student, student’s analysis.

	Sensitivity	Specificity	PPV	NPV	χ2	*p*-Value
FNA						
ChatGPT-I	61%	94%	85%	79%	χ2 (1) = 34.952	<0.001
ChatGPT-II	55%	87%	72%	76%	χ2 (1) = 20.532	<0.001
Student	97%	98%	97%	98%	χ2 (1) = 91.691	<0.001
Follow-Up						
ChatGPT-I	96%	43%	65%	91%	χ2 (1) = 21.830	<0.001
ChatGPT-II	98%	34%	63%	94%	χ2 (1) = 18.254	<0.001
Student	98%	96%	96%	98%	χ2 (1) = 88.341	<0.001

## Data Availability

Dataset available on request from the authors.
